# Structural and Biochemical Studies on ATP Binding and Hydrolysis by the *Escherichia coli* RNA Chaperone Hfq

**DOI:** 10.1371/journal.pone.0050892

**Published:** 2012-11-30

**Authors:** Hermann Hämmerle, Mads Beich-Frandsen, Branislav Večerek, Lukas Rajkowitsch, Oliviero Carugo, Kristina Djinović-Carugo, Udo Bläsi

**Affiliations:** 1 Department of Microbiology, Immunobiology and Genetics, Max F. Perutz Laboratories, University of Vienna, Vienna, Austria; 2 Department of Structural and Computational Biology, Max F. Perutz Laboratories, University of Vienna, Campus Vienna Biocenter 5, Vienna, Austria; 3 Institute of Microbiology, Academy of Sciences of the Czech Republic, Prague, Czech Republic; 4 Department of Biochemistry, Faculty of Chemistry and Chemical Technology, University of Ljubljana, Ljubljana, Slovenia; Max-Planck-Institute for Terrestrial Microbiology, Germany

## Abstract

In *Escherichia coli* the RNA chaperone Hfq is involved in riboregulation by assisting base-pairing between small regulatory RNAs (sRNAs) and mRNA targets. Several structural and biochemical studies revealed RNA binding sites on either surface of the donut shaped Hfq-hexamer. Whereas sRNAs are believed to contact preferentially the YKH motifs present on the proximal site, poly(A)_15_ and ADP were shown to bind to tripartite binding motifs (ARE) circularly positioned on the distal site. Hfq has been reported to bind and to hydrolyze ATP. Here, we present the crystal structure of a C-terminally truncated variant of *E. coli* Hfq (Hfq_65_) in complex with ATP, showing that it binds to the distal R-sites. In addition, we revisited the reported ATPase activity of full length Hfq purified to homogeneity. At variance with previous reports, no ATPase activity was observed for Hfq. In addition, FRET assays neither indicated an impact of ATP on annealing of two model oligoribonucleotides nor did the presence of ATP induce strand displacement. Moreover, ATP did not lead to destabilization of binary and ternary Hfq-RNA complexes, unless a vast stoichiometric excess of ATP was used. Taken together, these studies strongly suggest that ATP is dispensable for and does not interfere with Hfq-mediated RNA transactions.

## Introduction

The *Escherichia coli* host factor I/Q (Hfq) protein was first described as a factor required for replication of the RNA plus-strand of bacteriophage Qβ [1], [2]. Disruption of the *hfq* gene caused broad pleiotropic phenotypes, suggesting that Hfq plays a general role in *Escherichia coli* physiology [3]. More recently, Hfq was shown to mediate the interaction between *trans*-encoded small regulatory RNAs (sRNAs) and mRNAs, which can result in translational silencing or activation [4], [5]. In addition, Hfq binding to sRNAs and mRNAs can affect their stability [6].

Crystallographic studies of the conserved core of Hfq of different Bacteria revealed a hexameric ring-shaped structure, similar to Sm and Sm-like proteins involved in RNA transactions in Eukaryotes [7], [8], [9], [10]. Biochemical, genetic and structural studies suggested two distinct RNA binding sites on Hfq. The proximal site appears to preferably bind sRNAs, whereas the distal site binds A-rich motifs [11], [12]. The 3-D structure of the *Staphylococcus aureus* Hfq protein in complex with a single-stranded hepta-oligoribonucleotide (AU_5_G) showed that the RNA oligonucleotide was bound on the proximal site in a circular manner along the inner, basic rim of the central pore [7]. Link *et al*. [12] reported a crystal structure of three subunits of *Escherichia coli* Hfq in complex with nine adenines of poly(A_15_), with the poly(A) tract bound to the distal face of Hfq using tripartite binding motifs. They consist of (i) an adenosine specific site (A- site), which is a surface-exposed groove composed of residues from ß-strands 2 and 4, (ii) a purine nucleotide selective site (R-site), which is characterized by a crevice formed between the β-sheets of two neighbouring subunits and (iii) a sequence-non-discriminating E-site [12].

It has been reported that ATP binds to Hfq and that the protein possesses ATPase activity [13], [14]. Using molecular modelling Arluison *et al*. [14] proposed that the ATP-binding sites are located near the conserved Tyr-25 residue. Consistently, a Tyr-25Ala Hfq variant was impaired in ATP binding and displayed reduced ATP hydrolysis [14]. In addition, RNA footprinting and electrophoretic mobility shift assays indicated a dissociation of Hfq-RNA complexes in the presence of ATP [14]. Other biophysical studies showed that Hfq alone does not promote strand displacement of RNA oligonucleotides [15], and that the proposed ATPase activity of Hfq is not required for annealing [16]. Moreover, at variance with other *in silico* data [17] the 3D structure of the first 65 aa of *E. coli* Hfq in complex with ADP showed that ADP binds to the R-site at the distal face of the hexamer [18].

In this study, we revisited ATP binding in conjunction with the proposed ATPase activity of Hfq as well as a possible impact of ATP on annealing of complementary RNA oligonucleotides. The X-ray structure of the first 65 aa of *E. coli* Hfq in complex with ATP revealed that – like in the ADP bound form [18] – the ligand resides in the R-sites of the tripartite binding motif (ARE) on the distal face of Hfq hexamer. However, using full length Hfq protein purified to homogeneity we could not confirm the reported ATPase activity, nor did we observe a significant destabilization of Hfq-RNA complexes in the presence of ATP.

## Results and Discussion

### ATP Binding to the Distal R-site of Hfq

The crystals were obtained from a C-terminally truncated Hfq variant, comprising aa 1–65 of *E. coli* Hfq (Hfq_65_) in complex with ATP. The structure of the Hfq_65_–ATP complex was refined to 2.15 Å resolution with R_work_ and R_free_ values of 0.229 and 0.257, respectively. The data collection and refinement statistics are summarized in Table 1. The superposition of Hfq_65_-ATP with the apo-structure (PDBid: 1HK9) [8] exhibits an overall r.m.s.d. of 0.53 Å over 360 equivalent Cα atoms, indicating no significant conformational changes upon ATP binding.

**Table 1:** Data collection and refinement statistics.

Beamline	ID14.1 (ESRF)
Wavelength (Å)	0.934
Resolution (Å)^a^	98.67–2.15(2.21–2.15)
Space group	C2
Unit cell (Å,°)	a = 104.30b = 40.62c = 100.81β = 101.8
Molecules/a.u.	6
Unique reflections	21689 (1484)
Completeness (%)	99.81 (88.9)
R_meas_ *^b^*	0.079 (0.469)
R_merge_ *^c^*	0.111 (0.432)
Multiplicity	5.61 (5.62)
I/sig(I)	17.96 (3.70)
R_work_ *^d^*/R_free_ *^e^*	0.2294/0.2573
R.m.s.d. bonds (Å)	0.008
R.m.s.d. angles (°)	1.22

a Values in parentheses are for the highest resolution shell.

b
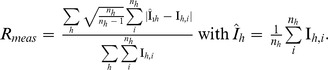

c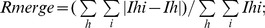 where I_i_ is the intensity of an individual reflection and <I> is the mean intensity of same reflection.

d R_work_ = Σ |F_o_-F_c_|/Σ F_o._

e R_free_ is the cross-validation R_factor_ computed for the test set of reflections (5%) which were omitted in the refinement process.

**Figure 1:**
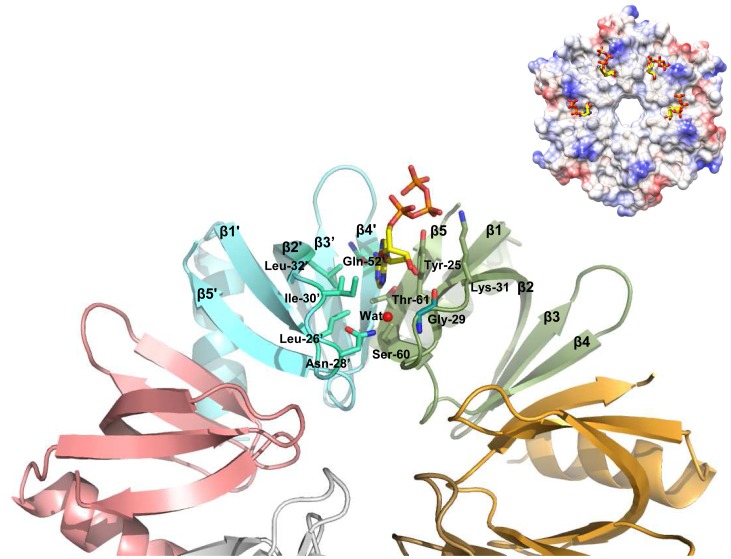
The purine binding site between adjacent subunits on the distal face of *E. coli* Hfq. Side-chains of binding site residues are shown by a stick. A single ATP-molecule is depicted with the triple-phosphate protruding in the favored conformation (transparent orange stick). Inset: The solvent accessible area of Hfq hexamer, colored according to its electrostatic potential (red and blue correspond to negatively and positively charged residues, respectively), is shown from the distal face with four ATP molecules bound.

In the present structure, electron density corresponding to the ATP molecules was observed in four out of six R-sites (inset in Figure 1). A single ATP molecule inserted into one R-site at the distal face of the Hfq_65_ hexamer is shown in Figure 1. The adenine moiety is stabilized in the binding pocket by aromatic stacking interaction with Tyr-25 and by favourable hydrophobic interactions with the side chains of Leu-26, Ile-30, and Leu-32 of the adjacent subunit. The adenine nitrogen atom N3 is hydrogen-bonded to a buried water molecule, which in turn hydrogen bonds to the main-chain carbonyl oxygen of Leu-26, and to the side-chains of Ser-60 and Asn-28. Similarly, N1 of the purine ring is hydrogen bonded to the side-chain of Thr-61, while N7 is hydrogen bonded to a solvent molecule in one out of three ATP molecules bound to the Hfq_65_ hexamer, while in the other three ligands N7 is not within hydrogen boding distance from solvent or protein atoms. In the latter three ATP molecules, the exocyclic N6 hydrogen bonds to the side-chain oxygen atom OE1 of Gln-52, with distances ranging from 2.66–3.26 Å, while in the fourth ligand, which is more deeply buried in the binding pocket, N6 hydrogen bonds to OG oxygen atom of Thr-61 (2.86 Å).

**Figure 2:**
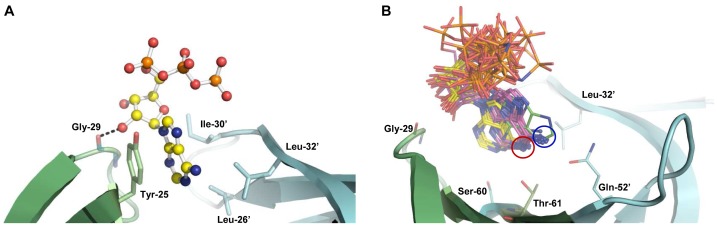
Interactions of adenines in Hfq-nucleotide complexes. (**A**) Hydrogen bond **i**nteraction between ribose 2′-OH with the Gly-29 carbonyl atom, for which a specific main chain conformation is required. ATP is shown as balls and sticks with the following color code for atoms: C – yellow; N - blue; O - red; P – orange. Hfq_65_ residues involved in the interaction are presented by a stick. (**B**) Superposition of Hfq_Bs_-AGAGAG (*B. subtilis* Hfq), Hfq-ADP (*E. coli* Hfq), Hfq-polyA (*E. coli* Hfq), Hfq_Pa_-ADPNP (*P. aeruginosa* Hfq) and Hfq_65_-ATP complexes to highlight the spread of purine ring orientations around the normal to the ring. Blue and red circles enclose two main clusters: in the first the exocyclic N6 atom hydrogen bonds to the Gln-52′ side-chain, in the second to the Thr-61 side-chain. N6 atoms are depicted as spheres. The colour code for atoms in the first cluster is: C - cyan; N - blue; O - red; P – orange; in the second C - yellow, and in the outlier ligand of the Hfq_Pa_-ADPNP complex C - green.

The comparison of the interactions of adenine in the complexes Hfq_Bs_-AGAGAG (*B. subtilis* Hfq), Hfq-ADP (*E. coli* Hfq), Hfq-polyA (*E. coli* Hfq), Hfq_Pa_-ADPNP (*P. aeruginosa* Hfq) [12], [17], [18] (PDBids: 3HSB, 3RES, 3GIB, 3QUI, respectively) with the Hfq_65_-ATP complex shows that the purine moiety in the binding pocket can adopt variable positions. While the angles of the aromatic rings of adenine with the plane of the Tyr-25 side-chain are precisely defined *via* π…π stacking interactions on one side of the binding pocket, and by hydrophobic interactions on its opposite side (Figure 2A), a rotation of the purine ring around the normal to the ring plane can be observed. This leads to a spread of orientations with concomitant difference in depth of the ligand in the pocket (Figure 2B). Apart from the position of one ligand in the Hfq_Pa_-ADPNP complex, the ligand positions can be clustered in two groups.

In the first cluster (composed of ligands in the Hfq_Bs_-AGAGAG complex, and part of the ligands in Hfq_Ec_-ADP-AU6A and Hfq_Ec_-ADP complexes), N6 is hydrogen bonded to the side-chain of Thr-61, while N1 is at hydrogen bonding distance to Ser-60. In the second cluster (composed of ligands in Hfq_Pa_-ADPNP, Hfq_Ec_-polyA, and part of the ligands in the Hfq_Ec_-ADP-AU6A and Hfq_Ec_-ADP complexes), the exocyclic N6 is hydrogen bonded to the side-chain of Gln-52′ and N1 to the side chain of Thr-61. In essence, a unique binding position of ligands is observed in the case of Hfq_Pa_-ADPNP, Hfq_Ec_-polyA, and Hfq_Bs_-AGAGAG complexes, while in the case of Hfq_Ec_-ADP-AU6A and Hfq_Ec_-ADP the ligands adopt both binding modes. In our crystal structure three out of four ATP ligands bind according to the second cluster binding mode, and one according to the first.

Common to all the binding sites is the coordination of the adenosine ribose 2′-OH by the carbonyl-group of Gly-29 (Figure 2A). This coordination was suggested to convey selectivity for RNA over DNA for this binding site [12]. Our results corroborate the importance of coordinating the 2′-OH of the ribose sugar, through the Gly-29 carbonyl oxygen atom. With its φ/ψ-angles falling in the region around ∼85° and ∼−10°, respectively, Gly-29 adopts main-chain conformation allowed only for residues, which do not posses a side-chain. This suggests that any substitution of Gly-29 will dramatically reduce the capacity of Hfq to bind an adenosine ligand in the R-site, preventing the formation of this interaction by changing the main-chain conformation, and therefore a favourable position of the Gly-29 carbonyl oxygen for formation of this hydrogen bond.

The electron density for the three-phosphates, which was weaker compared to the rest of the ligand, revealed different conformations of the phosphates, which bind with their first phosphate group either directly or *via* solvent-mediated interactions to the hydroxyl group of Tyr-25 and the NZ atom of Lys-31 (Figure 1), while the second and the third phosphate groups are solvent exposed. Similarly, a spread of tri-phosphate conformations was also observed in other Hfq-adenosine complexes [12], [18], [19] (Figure 2A, B).Thus, our interpretation is that the tri-phosphates do not have a specific conformation, and are tethered to the protein only *via* the first phosphate group.

A modified Walker-A motif (-GXXXXGKT-) [20] - known as a common nucleotide binding motif - was proposed for the sub-sequence -Gly29IKLQGQI36-, residing on the distal face of the Hfq hexamer. The Walker-A motif typically adopts a loop conformation and utilizes the highly conserved residues lysine and threonine to bind to the phosphate-oxygen atoms in the presence of a magnesium ion, which coordinates the β- and γ-phosphates. In the Hfq-ATP complex, Gly-29 interacts specifically with the adenosine ribose 2′-OH and not with the phosphate groups, which in turn bind to Tyr-25 and Lys-31, making it rather unlikely that Hfq is endowed with ATPase activity. The comparison of the Hfq-ATP model proposed by Arluison *et a.* [14] with our experimental structure reveals that in the model the adenosine ring is flipped by 180°, with the exocyclic N6 atom pointing upwards, as well as rotated by 180°, leading to a different conformation and interactions of the tri-phosphate moiety.

### Hfq is Devoid of ATPase Activity

**Figure 3:**
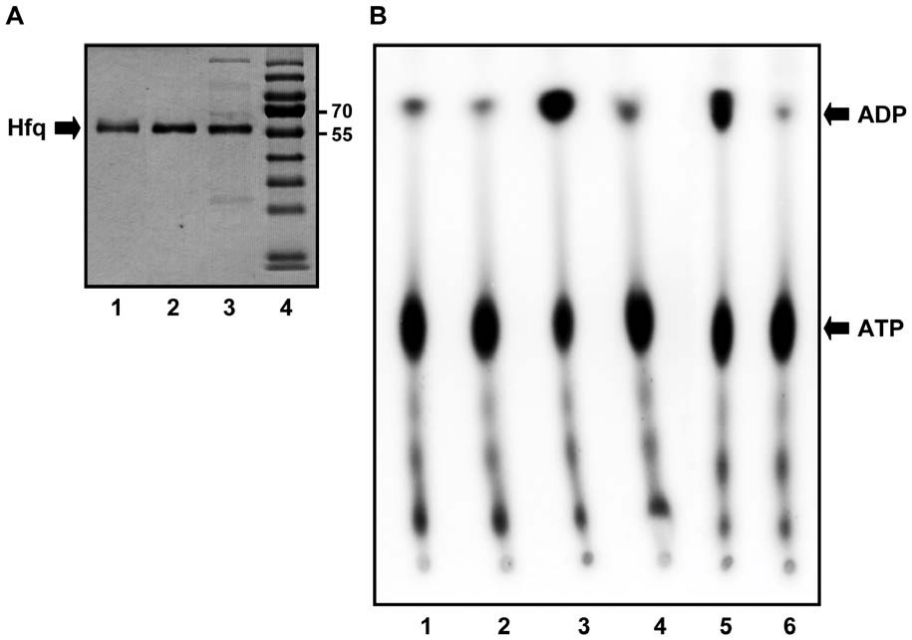
ATPase activity assays. (**A**) Analysis of different Hfq preparations after electrophoresis on a 12% SDS–polyacrylamide gel. Lane 1, heat-treated Hfq preparation #1. Lane 2, non-heated HIC-purified Hfq preparation #3. Lane 3, non-heated Hfq preparation #2. Lane 4, molecular masses of selected marker proteins (in kD). The position of the Hfq-hexamer is indicated by an arrowhead. (**B**) Thin layer chromatography/ATPase activity assays. Lane 1, no protein added; Lane 2, Hfq preparation #1; Lane 3, Hfq preparation #2; Lane 4, Hfq preparation #2 heated to 85°C; Lane 5, mock preparation obtained from the *hfq-* strain JW4130; Lane 6, non-heated HIC-purified Hfq preparation #3.

The reported ATPase activity of Hfq [13], [14] could have biological relevance with regard to Hfq-mediated RNA transactions. We therefore revisited the ATPase activity by using full length Hfq protein prepared by different procedures. First, full length *E. coli* Hfq was purified following a protocol that comprises a heating step at 85°C to remove mesophilic proteins. As recently described in detail [21], this procedure (Hfq preparation #1) included Ni^2+^-affinity purification followed by anion-exchange chromatography. As a single subunit of the protein harbours four histidines at the C-terminus, Ni^2+^-affinity purification was performed without recombinant addition of histidines to the protein. As judged by SDS-gel electrophoresis, this Hfq preparation did not contain visible contaminant proteins after PageBlue staining (Figure 3A, lane 1). Hfq protein purified in this manner did not display ATPase activity (Figure 3B, lane 2). As full length *E. coli* Hfq protein purified according this protocol was proficient in RNA binding and annealing activity [22], we next tested whether the heating step could have led to inactivation of the putative ATPase activity of Hfq.

Hfq was again purified using Ni^2+^-affinity purification, this time omitting the heating step. This procedure yielded a Hfq preparation (#2) of moderate purity (Figure 3A, lane 3), which displayed ATPase activity (Figure 3B, lane 3). After heat-treatment at 85°C for 15 min of Hfq preparation #2, an ATPase activity was no longer observed (Figure 3B, lane 4). Therefore, the non-heat treated Hfq preparation #2 was further purified using hydrophobic interaction chromatography. The protein preparation was loaded onto a Butyl-FF column yielding a Hfq preparation (#3) of high purity (Figure 3A, lane 2). Hfq preparation # 3 did not display ATPase activity (Figure 3B, lane 6). The latter experiment argued against the possibility that the absence of an ATPase activity observed with Hfq preparation #1 resulted from heat inactivation. In addition, a mock preparation obtained from the *hfq* deletion strain JW4130 with protocol #2 was proficient in ATP hydrolysis (Figure 3B, lane 5), again demonstrating that the ATPase activity obtained with Hfq preparation #2 can not be attributed to Hfq.

As mentioned above, the lack of an ATPase activity of Hfq can be rationalized from the structure of the Hfq_65_-ATP complex. The structural data presented above would argue against an ATPase activity of Hfq for at least two reasons. First, ATP was apparently not hydrolysed in the Hfq_65_-ATP complexes. Second, the triphosphate of the ATP molecule points away from the putative Walker-A motif -G_29_IKLQGQI_36_- which comprises a substantial part of the β4 strand and does not adopt a loop structure in Hfq. Furthermore, only the α-phosphate interacts with Tyr-25 and Lys-31 side-chains in a direct or solvent mediated manner (Fig. 1), while β- and γ- phosphates remain solvent exposed.

Our data are at variance with the reported ATPase activity of Hfq [13], [14]. Sukhodolets and Garges [13] reported that the ATPase activity was retained in the Hfq preparation upon heating a cell lysate to 80°C. The Hfq purification protocol used here [21] included a heating step at 85°C for 45 minutes. Thus, we can only speculate that the heating step used in [13] was not sufficient to inactivate contaminating ATPase(s). In addition, here we have heat-treated the highly enriched Hfq preparation #2 (Fig. 3A) for 15 min at 85°C. Hence it is also possible that heating a lysate to 80°C rather than an enriched fraction does not inactivate all contaminating ATPase. On the other hand, as a heating step was omitted in the study by Arluison *et al.* [14] (V. Arluison, personal communication) the reported ATPase of Hfq likely resulted from impurities co-purifying with Hfq as observed here for preparation #2. However, these authors also reported a strongly reduced ATPase activity for a HfqY25A mutant. As this residue is instrumental for ATP binding in the R-site (Figure 1 and 2), this finding is difficult to reconcile with the absence of any notable ATPase activity in Hfq preparations #1 and #3. Nevertheless, the formal possibility of an association of the more hydrophopic distal surface of Hfq versus HfqY25A with an ATPase could reconcile the observations.

### ATP does not Affect Annealing of RNA Ligands by Hfq

**Figure 4:**
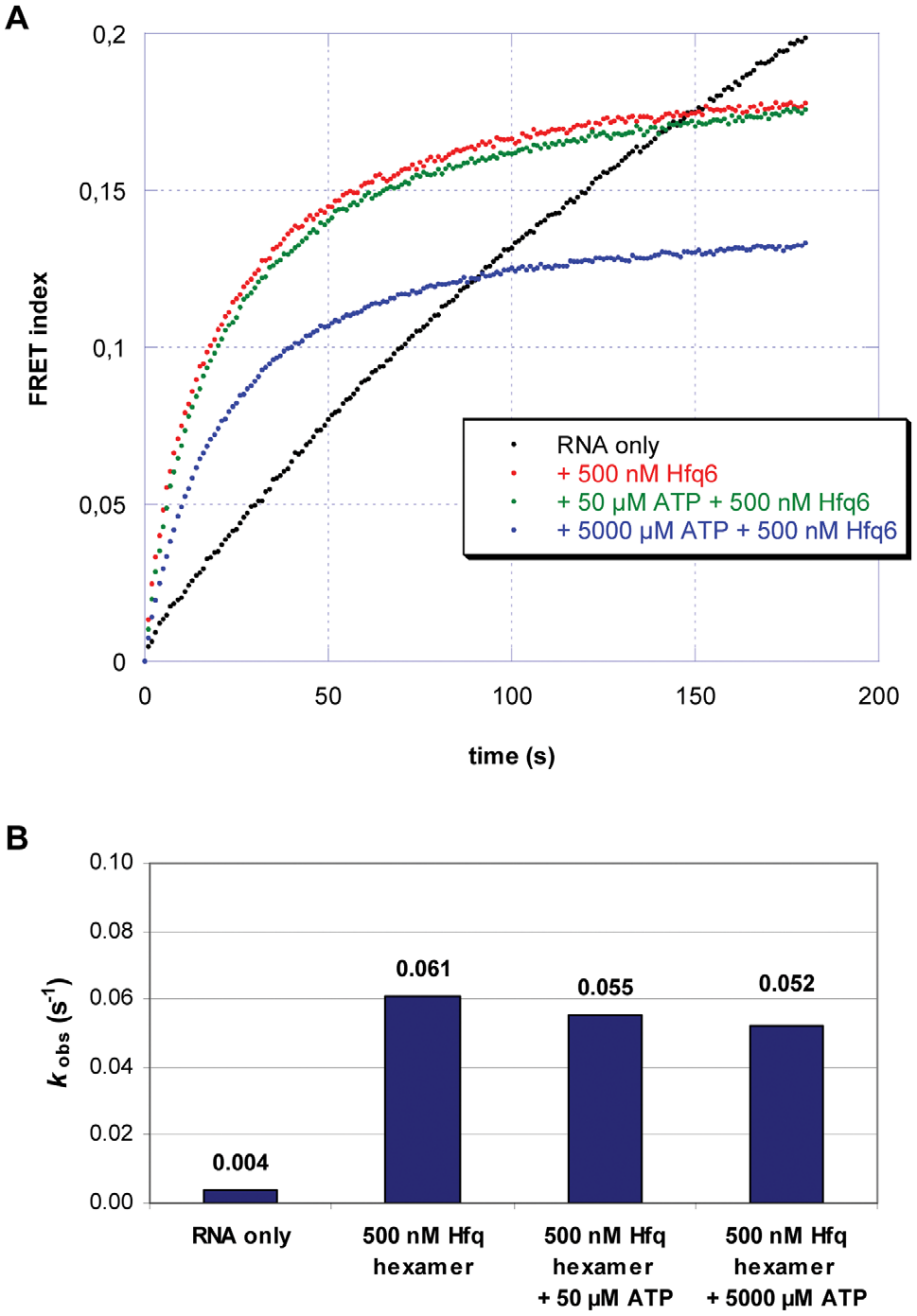
RNA annealing activity of Hfq is ATP-independent. (**A**) 5 nM of two complementary RNAs (Cy5–21R^+^, Cy3–21R^−^) were annealed in a microplate reader in the absence or presence of Hfq at 37°C in 50 mM Tris-HCl, 3 mM MgCl_2_ and 1 mM DTT in the presence of different ATP concentrations. The donor (Cy3) and acceptor (Cy5) fluorescence emissions were quantified every second. The FRET index was calculated as F_Cy5_/F_Cy3_. The curves were least-square fitted with the second-order reaction equation for equimolar initial reactant concentrations: P_t_ = P·(1−1/(*k*_obs_·t+1)); P_t_ = fraction annealed, P = max. FRET index. Note that the amplitude of the FRET index is only indicative and does not correspond to the absolute percentage of double-stranded RNAs. (**B**) Reaction constants for RNA annealing at different ATP concentrations.

Hfq was previously shown to stimulate duplex formation of complementary RNA ligands [15], [16], [22], [23]. To test whether ATP could influence the annealing function of Hfq, fluorescence resonance energy transfer assays (FRET) were performed. Two complementary RNA-oligonucleotides, Cy5-21R^+^ and Cy3-21R^−^ (see Materials and Methods) were labeled at their 5′-ends with Cy5 and Cy3, respectively, and duplex formation was assessed in the absence and presence of ATP. Hfq stimulated annealing of these RNA-oligonucleotides with a rate constant *k_ann_* of 0.061 s^−1^, which was ∼ 15-fold higher than observed for self-annealing of the two RNA-oligonucleotides (Figure 4). A 100-fold and a 10.000-fold molar excess of ATP over the Hfq hexamer did not significantly affect annealing (Figure 4).

The ribonucleotide Cy5-21R^+^ contains a stretch of consecutive adenine residues and can therefore potentially bind to the distal face of Hfq [12]. Hfq hexamers bind to Cy5-21R^+^ with a k_d_ of ∼115 nM [22], whereas the k_d_ for ATP was determined with ∼125 nM [14], which could explain why even a molar excess of ATP over Hfq did not significantly affect annealing.

### ATP does not Destabilize Binary and Ternary Hfq-RNA Complexes

**Figure 5:**
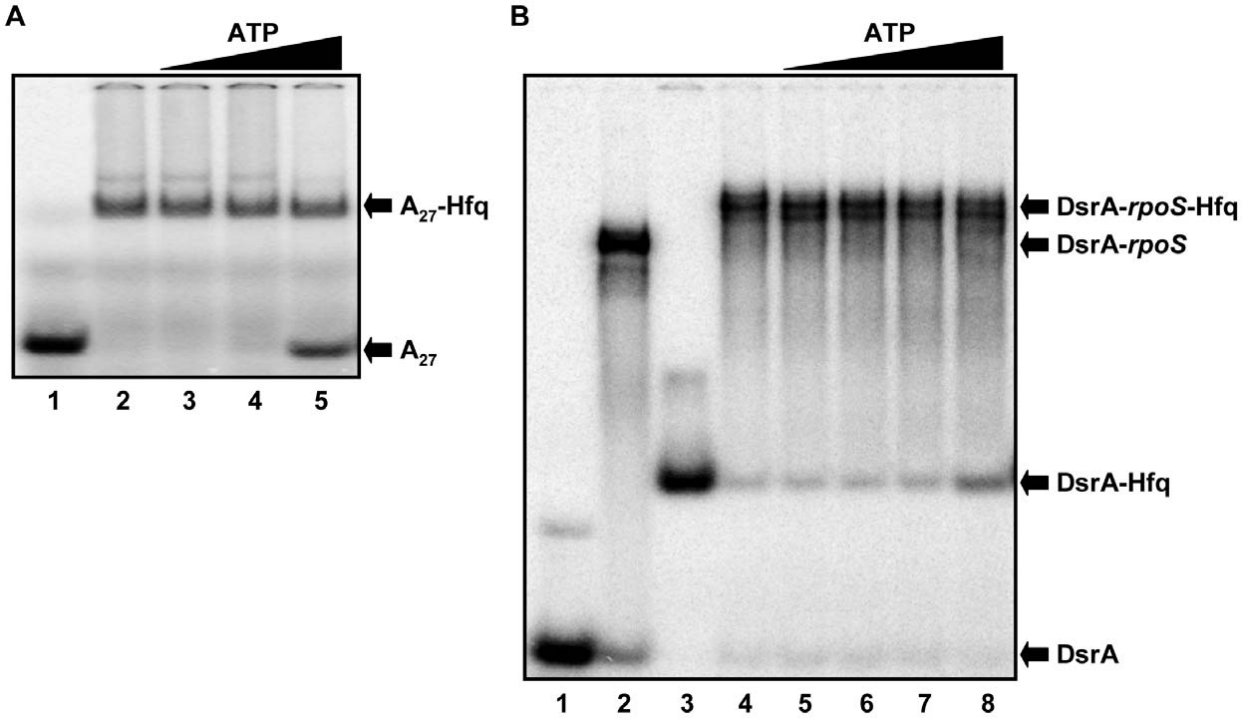
Influence of ATP on Hfq-RNA complexes. (**A**) Full length *E. coli* Hfq was added to poly(A)_27_ RNA to form stable complexes, which were subsequently challenged by addition of increasing amounts of ATP. 5′-end labelled poly(A)_27_ RNA (5 nM) was incubated in the absence (lane 1) or in the presence of 40 nM (lanes 2–5) Hfq-hexamer. 0.1 mM (lane 3), 1 mM (lane 4) and 10 mM (lane 5) ATP was added as competitor. (**B**) Ternary complexes between full length *E. coli* Hfq, DsrA and *rpoS_652_* RNA were challenged with ATP. 5′-end labelled DsrA (5 nM) was incubated in the absence (lane 1 and 2) or in the presence of 160 nM Hfq-hexamer (lanes 3–8) and in the absence (lanes 1 and 3) or presence of 50 nM *rpoS_652_* mRNA (lane 2 and lanes 4–8). 10 µM (lane 5), 100 µM (lane 6), 1 mM (lane 7) and 10 mM (lane 8) ATP was added after ternary complex formation.

ATP was suggested to destabilize Hfq-RNA complexes [14]. Thus, ATP could potentially act as a competitor *in vivo* and promote dissociation of Hfq from RNA when ATP levels are sufficiently high, i.e. under conditions of optimal growth. Therefore increasing amounts of ATP were added to pre-formed Hfq-poly(A)_27_ complexes (Figure 5A). Only at the highest ATP concentration (10 mM; 2×10^6^-fold stoichiometric excess of ATP over poly(A)_27_) approximately 50% of the RNA was released from Hfq.

Next, we asked whether ATP is able to promote dissociation of ternary complexes formed between Hfq and its natural ligands, the *E. coli* sRNA DsrA and *rpoS* mRNA, encoding σ^S^. In this experiment a 652 nucleotide long fragment (*rpoS*_652_) of *rpoS* mRNA comprising the complete 5′-UTR of *rpoS* was used. Again, *rpoS*_652_ was only released from the ternary complex when a vast excess of ATP was added (Figure 5B). This result is in agreement with the known binding specificity of Hfq for *rpoS*_652_ and DsrA. While DsrA is known to bind to the proximal site of Hfq [11], the 5′UTR of *rpoS* mRNA contains adenine rich binding motifs that bind to the distal side of Hfq [24], [25]. Thus, as the band intensity for the Hfq-DsrA complex increased upon addition of high excess of ATP (Figure 5B), *rpoS*_652_ was apparently replaced by ATP. However, given the vast excess of ATP required to displace ∼10% of *rpoS*_652_ from the ternary complex we do not consider this result as being physiologically relevant. In addition, the affinity of Hfq for ATP [14] is ∼ 1 and 2 orders of magnitude lower than for the 5′UTR of *rpoS* mRNA and for the poly(A)_27_ oligoribonucleotide [12], respectively. Although the approximate intracellular stoichiometries of Hfq_6_ : ATP is in the range of 1∶1000 [26], [27], the competition experiments would argue against an interference of ATP with the binding of RNA substrates to the distal site of Hfq.

## Materials and Methods

### Bacterial Strains and Growth Conditions

The *E. coli hfq^−^* strain AM111 [28], the corresponding F’ (*lacI^q^*) variant AM111F’ [29] and the *hfq* deletion strain JW4130 [30] have been described. They were grown in Luria-Bertani medium at 37°C. Kanamycin (25 µg/ml) and ampicillin (100 µg/ml) were added where appropriate.

### Hfq/Hfq_65_ Purification

For crystallization, the C-terminally truncated *E. coli* Hfq protein, Hfq_65_, was purified from strain AM111F’ harbouring plasmid pUHfq65 [22]. Hfq synthesis was induced at an OD_600_ of 0.6 by addition of isopropyl β-D-1-thiogalactopyranoside (IPTG; 0.5 mM final concentration). After 3 hours, the cells were harvested by centrifugation at 4000×g for 15 min. Lysis was achieved by a French-press at 4°C in 50 mM Tris-HCl pH 7.4, 1.5 M NaCl, 250 mM MgCl_2_, 1 mM phenylmethanesulfonylfluoride (PMSF), 0.5 mM β-mercaptoethanol. After lysis, 25 µg/ml of DNase I (Sigma-Aldrich) was added for 20 min and the lysis-solution was cleared by centrifugation at 45.000×g for 15 min. Further purification steps included heat-fractionation followed by a series of FPLC-based chromatographic steps, hydrophobic interaction chromatography (HIC) and anion-exchange chromatography (aIEX) as recently described in detail [21]. The purified protein was concentrated in Centricon spin-filters (Amicon) to 15 mg/ml in a buffer containing 50 mM Tris-HCl pH 7.4, 200 mM NaCl.

For the ATPase activity assays, full length *E. coli* Hfq was purified using the protocol outlined in [21]. *E. coli* strain AM111F’ bearing plasmid pUH5 [31] and strain JW4130 (mock preparation) were grown at 37°C in LB medium. At an OD_600_ of 0.6, IPTG was added to a final concentration of 0.5 mM to induce synthesis of the Hfq protein. After 3 hours the cells were harvested by centrifugation (5000 rpm; 4°C). Hfq preparation #1 was obtained after heating of the lysate to 85°C prior to Ni^2+^-affinity chromatography and aIEX chromatography [21]. For Hfq preparations #2 and #3 the pellet was resuspended in chilled lysis buffer (50 mM Tris-HCl pH 8, 1.5 M NaCl, 250 mM MgCl_2_, 5 mM DTT, 1 mM PMSF) and the cells were lysed by sonication. Then, Ni^2+^-affinity chromatography was performed followed by dialysis overnight at 4°C in 1 L GF buffer (50 mM Tris-HCl pH 8.0, 200 mM NaCl) using benzoylated dialysis tubes (Sigma-Aldrich). This procedure yielded Hfq preparation #2, which was analyzed on a 12% SDS–polyacrylamide gel using PageBlue protein staining (Fermentas). For further purification of Hfq preparation #2, the salt concentration was adjusted to 4 M NaCl and the protein sample was loaded onto a 1 ml HiTrap Butyl-FF column (GE healthcare), equilibrated with buffer (50 mM Tris-HCl pH 8, 4 M NaCl), and washed extensively for ∼ 30 column volumes with the same buffer. Elution was achieved by lowering the salt-concentration by stepwise switching to a buffer containing 50 mM Tris-HCl pH 8, 200 mM NaCl (GF buffer). This procedure resulted in Hfq preparation #3, which was dialyzed overnight at 4°C in 1 L GF buffer and again analyzed on a 12% SDS–polyacrylamide gel.

### Crystallization and Data-collection

Prior to crystallization the protein was mixed with adenosine-triphosphate (ATP; Sigma-Aldrich) in a 1∶1 molar-ratio with respect to one subunit of Hfq_65_. Nano-litre crystallization trials using the JSCG+-screen [32] were set by a robot in 96-well vapor diffusion sitting-drop format. Diffraction quality crystals were obtained directly from the JCSG+-screen, and the data were collected from a crystal grown in 0.1 M Hepes pH ∼ 7.5, 10% (w/v) PEG-8000 and 8% (v/v) ethylene glycol.

The crystals were harvested using cryo-loops of suitable size (Hampton) and frozen in liquid nitrogen without usage of a cryo-protectant. A complete data-set was collected at the beamline ID14.1 at the European Synchrotron Facility (ESRF), and processed with the XDS-suite [33] in space group C2, to a maximum resolution of 2.15 Å. Data-collection and refinement statistics are outlined in Table I.

### Structure Determination and Refinement

The structure was determined by molecular replacement, using Molrep [34] from the CCP4-suite [35], with one Hfq monomer-chain from the Hfq-hexamer as search-model (PDBid: 1HK9, aa 5–65) [8]. The rotation function had six distinct solutions, corresponding each to one of the subunits in the hexamer. Refinement was performed by phenix.refine in the Phenix-suite [36] employing simulating annealing and energy minimization cycles. Initially tight geometry restraints were applied, releasing them gradually as the R and R_free_ factors diverged. After several rounds of iterative refinement and model building with COOT [37], a clear density for ATP-molecules was found in four of six binding-sites. Addition of ATP to the model (with occupancy fixed at 1) caused the R and R_free_ factors to converge, and refinement was continued with increasing restraints, until no significant change in R-factors was obtained. A final step of refinement by Refmac [38], [39] in the CCP4-suite [35], was done to adjust the weighting between X-ray and stereochemical terms for minimizing the R-factor. Final validation of the models was performed with MOLPROBITY [40].

### Structural Analysis and Structural Comparisons

Structural comparison and superposition was done in Pymol [41]. Pymol was also used for the generation of Figures. The analysis of the R-site binding pocket was performed using the Protorp server (http://www.bioinformatics.sussex.ac.uk/protorp/) [42]. The model coordinates and structure factors were deposited in the PDB under code 3QO3.

### ATPase Activity Assays

The ATPase activity assays were performed as described [13] in 15 µl reaction buffer (50 mM Tris-HCl pH 7.5, 100 mM NaCl, 2 mM MgCl_2_) containing 0.25 µg of purified Hfq (∼ 4 pmol Hfq hexamers), 0.1 mM ATP, 0.5 µCi of [α-^32^P]-ATP. The reaction mixtures were incubated for 90 min at 37°C. Then, 4 µl of the samples were spotted onto poly(ethylenimine)-cellulose plates (Merck TLC PEI cellulose F). The chromatography was carried out in 1 M LiCl, 1 M formic acid and ATP hydrolysis was visualized using a PhosphorImager (Molecular Dynamics).

### RNA Preparation for *in vitro* Studies

Poly(A)_27_ RNA was purchased (Darmacon) and gel-purified on a 8% polyacrylamide-8M urea gel according to standard protocols. The sRNA DsrA and *rpoS_652_* mRNA, comprising the full length 5′-UTR of *rpoS* mRNA were *in vitro* transcribed and gel-purified as described [22].

### Electrophoretic Mobility Shift Assays (EMSA)

Gel-purified RNAs were 5′-end labelled with [γ-^32^P]-ATP (Amersham Pharmacia Biotech) and re-purified on 6% polyacrylamide-8M urea gels. Labeled RNAs (5 nM) were incubated without or with increasing amounts of purified Hfq protein (as indicated in the legend to Figure 5A and B) and unlabelled *rpoS_652_* mRNA (where indicated) in a 10 µL reaction in binding buffer (10 mM Tris at pH 7.5, 60 mM NH_4_Cl, 5 mM β-mercaptoethanol, 2 mM MgOAc, 100 ng of yeast tRNA) for 10 min at room temperature (RT). Then, increasing amounts of ATP (as indicated in the legend to Figure 5A and B) were added to the samples and the incubation was continued for 10 min at RT. The samples were mixed with 40% glycerol to a final concentration of 10% and loaded on a native 4% polyacrylamide gel. Electrophoresis was performed in TAE buffer (40 mM Tris pH 8, 20 mM acetic acid, 1 mM EDTA) at 140 V for 2–6 hrs. Radioactive bands were visualized using a PhosphorImager (Molecular Dynamics).

### FRET Assays

Experimental details on the FRET assays have been described [43], [44]. Briefly, two complementary, fluorophore-tagged RNA 21mers (Cy5-5′-AUGUGGAAAAUCUCUAGCAGU-3′ (Cy5-21R^+^) and Cy3-5′-ACUGCUAGAGAUUUUCCACAU-3′ (Cy3-21R^−^) were used in the experiment shown in Figure 4. The tagged RNA oligonucleotides were purchased from VBC-Biotech (Vienna, Austria). Using a Tecan GENios Pro microplate reader, the first oligoribonucleotide was injected into the wells with or without Hfq protein (500 nM final Hfq-hexamer concentration) or different concentrations of ATP, and the measurement was started with the injection of the second oligoribonucleotide. The reaction was performed in annealing buffer (50 mM Tris-HCl pH 7.5, 3 mM MgCl_2_ and 1 mM DTT) at 37°C. The final concentration of the RNAs was 5 nM in a volume of 40 µl. The reaction was allowed to proceed for 180 seconds, and with Cy3 excited, donor and acceptor dye fluorescence emissions were measured once every second. The time-resolved ratio of the fluorescence emissions (FRET index F_Cy5_/F_Cy3_) was normalized to 1 at t_180s_ and least-square fitted with Prism 4.03 (GraphPad Software Inc., San Diego, CA, USA) with the second-order reaction equation for equimolar initial reactant concentrations y = A (1–1/(*k_ann_* t +1)); *k_ann_* = observed annealing reaction constant, A = maximum reaction amplitude. The curves shown are representative; the observed reaction constants *k*_ann_ were calculated as the average of three individually fitted reactions.
